# Effects of Trunk Motion, Touch, and Articulation on Upper-Limb Velocities and on Joint Contribution to Endpoint Velocities During the Production of Loud Piano Tones

**DOI:** 10.3389/fpsyg.2020.01159

**Published:** 2020-06-10

**Authors:** Felipe Verdugo, Justine Pelletier, Benjamin Michaud, Caroline Traube, Mickaël Begon

**Affiliations:** ^1^Laboratoire de Simulation et Modélisation du Mouvement, Faculté de Médecine, École de Kinésiologie et des Sciences de L’activité Physique, Université de Montréal, Montreal, QC, Canada; ^2^Input Devices and Music Interaction Laboratory, Schulich School of Music, McGill University, Montreal, QC, Canada; ^3^Centre for Interdisciplinary Research in Music Media and Technology, Schulich School of Music, McGill University, Montreal, QC, Canada; ^4^Laboratoire de Recherche sur le Geste Musicien, Faculté de Musique, Université de Montréal, Montreal, QC, Canada; ^5^CHU Sainte-Justine Research Center, Montreal, QC, Canada

**Keywords:** piano performance, trunk motion, touch, articulation, biomechanics, inverse kinematics

## Abstract

Piano performance involves several levels of motor abundancy. Identification of kinematic strategies that enhance performance and reduce risks of practice-related musculoskeletal disorders (PRMD) represents an important research topic since more than half of professional pianists might suffer from PRMD during their career. Studies in biomechanics have highlighted the benefits of using proximal upper-limb joints to reduce the load on distal segments by effectively creating velocity and force at the finger–key interaction. If scientific research has documented postural and expressive features of pianists’ trunk motion, there is currently a lack of scientific evidence assessing the role of trunk motion in sound production and in injury prevention. We address this gap by integrating motion of the pelvis and thorax in the analysis of both upper-limb linear velocities and joint angular contribution to endpoint velocities. Specifically, this study aims to assess kinematic features of different types of touch and articulation and the impact of trunk motion on these features. Twelve pianists performed repetitive loud and slow-paced keystrokes. They were asked to vary (i) body implication (use of trunk and upper-limb motion or use of only upper-limb motion), (ii) touch (struck touch, initiating the attack with the fingertip at a certain distance from the key surface, or pressed touch, initiating the attack with the fingertip in contact with the key surface), and (iii) articulation (*staccato*, short finger–key contact time, or *tenuto*, sustained finger–key contact time). Data were collected using a 3D motion capture system and a sound recording device. Results show that body implication, touch, and articulation modified kinematic features of loud keystrokes, which exhibited not only downward but also important forward segmental velocities (particularly in pressed touch and *staccato* articulation). Pelvic anterior rotation had a prominent role in the production of loud tones as it effectively contributed to creating forward linear velocities at the upper limb. The reported findings have implications for the performance, teaching, and research domains since they provide evidence of how pianists’ trunk motion can actively contribute to the sound production and might not only be associated with either postural or expressive features.

## Introduction

Piano performance involves several levels of motor abundancy (Latash, 2012). Kinematic abundancy allows pianists to produce similar piano tones through an unlimited number of spatiotemporal motion profile possibilities across all joints of the kinematic chain. During the last century, diverse approaches to piano performance have emerged and currently coexist, each of them bringing out the musical, physiological, and mechanical advantages and disadvantages of distinct kinematic strategies (Kochevitsky, 1995). Despite this documented diversity, most artistic and scientific sources dealing with movement efficiency and proficiency in piano performance find a common ground in their focus on motion of upper-limb segments. Therefore, pianists’ trunk motion (i.e., pelvis and thorax motion) is either not addressed or remains associated with a postural role in at least three types of literature: (i) mainstream approaches to piano performance (e.g., Neuhaus, 1978; Gieseking and Leimer, 2013), (ii) studies in pianists’ motor behavior [see, e.g., Goebl (2017) for a recent review], and (iii) performing arts medicine literature (e.g., Bros and Papillon, 2001; Llobet, 2017). Fine control of upper-limb joint motion is indeed a fundamental aspect of highly skilled piano performance. However, pelvo-thoracic joints are also a part of the kinematic chain while striking the keys. Kinematic, kinetic, and physiological features of athletes’ and workers’ trunk motion have been addressed in studies on sports and on repetitive tasks in either standing (e.g., Wang et al., 2010; Reid et al., 2013; Côté, 2014) or sitting (e.g., Begon et al., 2010; Cavedon et al., 2014) positions. Overall, these studies suggest that trunk motion can actively contribute to the generation of velocity and force at the distal end of the kinematic chain. This goal-oriented use of trunk motion might enhance specific performance outcomes (e.g., energy transfer while hitting a ball) and decrease risks of injuries by, for instance, increasing inter-joint coordination possibilities to adapt to distal muscle fatigue (Côté et al., 2008) and reducing stress on distal joints and muscles (e.g., Seroyer et al., 2010; Oyama, 2012). A similar rationale might be applied to piano performance, as it is possible to hypothesize that the pelvis and the thorax could have a relevant impact on upper-limb segments’ velocities and on force generation at the finger–key interaction. This idea has been advanced in alternative approaches to piano performance such as the approach developed and thought at Université de Montréal, which encourages a systematic and active implication of specific pelvic and thoracic movements while performing (Verdugo, 2018; Verdugo et al., 2019). If scientific research has documented expressive and communicative features of pianists’ trunk motion (Thompson and Luck, 2012; Massie-Laberge et al., 2019), there is currently a lack of empirical evidence assessing the role of trunk motion in pianists’ sound production and injury prevention strategies.

Due to the long-lasting and repetitive character of professional instrumental practice, more than half of professional pianists might suffer from playing-related musculoskeletal disorders (PRMD) during their career (Kaur and Singh, 2016). In addition, a stronger incidence of PRMD has been found at the level of distal muscles and tendons (Bragge et al., 2006). Assessing this problem, researchers in biomechanics have shown that pianists might take advantage of a multi-joint motion of the upper limbs to efficiently create force at the finger–key interface and reduce the load on distal muscles (Furuya and Altenmuller, 2013). Finger–key interaction, usually referred to as “touch,” is nevertheless influenced not only by biomechanical constraints but also by expressive and accuracy needs. This explains why touch has been a recurrent subject of debate among pianists, pedagogues, and scientists (Goebl et al., 2014; MacRitchie, 2015; MacRitchie and McPherson, 2015). The study of two types of piano touch has predominated in scientific research: (i) struck touch (initiated with the fingertip at a certain distance from the key surface) and (ii) pressed touch (initiated with the fingertip in contact with the key surface). From a biomechanical standpoint, struck touch has been defined as a more efficient keystroke strategy because it enables a greater maximum key acceleration when producing loud tones (Kinoshita et al., 2007) and it facilitates an effective utilization of proximal-to-distal upper-limb intersegmental dynamics (Furuya et al., 2010). From an acoustical and sound-control point of view, pressed touch is recognized as a more effective strategy of tone production as it allows a better control of the piano mechanism during the entire key descent (Goebl et al., 2005) and helps enhance temporal accuracy between consecutive tones (Goebl and Palmer, 2008). Despite this dichotomy between pressed and struck touch, sources mentioned above acknowledge that pianists might rely on both types of touch to successfully adapt to the wide variety of musical contexts found in the piano repertoire. In this sense, our article presents specific features of struck and pressed touch and investigates how pelvo-thoracic joint motion might affect these features.

In addition to touch, pianists can control several musical parameters in order to shape the expressive content of their performance (e.g., articulation, timing, and dynamics). These parameters can be modulated in the context of both actual musical pieces or excerpts (Palmer, 1996; Bresin and Umberto Battel, 2000; Goebl, 2001; Bernays and Traube, 2014) and isolated piano tones (Baraldi et al., 2006). Since there is little scientific background on the impact of trunk motion on pianists’ kinematics, the present article focuses on a rather simple performance task: slow-paced and loud isolated piano tones. Several reasons support this choice. First, isolated tones might help reduce kinematic differences between pianists that would be linked to the expressive content of the excerpts performed. Second, slow-paced tones might facilitate the integration of a distinct trunk motion cycle to produce each isolated tone (on the contrary, the production of fast tones might result in the use of one single trunk motion encompassing several keystrokes). Third, as differences between struck and pressed touch are more salient in *forte* and *fortissimo* dynamics (Furuya et al., 2010), loud tones might be a better starting point to study the effects of trunk motion in both types of touch. Studies on motor behavior dealing with pressed and struck touch focus generally on short finger–key contact time, i.e., *staccato* articulation. To account for a wider variety of keystroke motion possibilities, this article integrates two types of articulation: *staccato* tones and *tenuto* tones (sustained finger–key contact time).

By studying pianists’ joint motion from the pelvis to the fingertip in the context of different types of touch (pressed and struck) and articulation (*staccato* and *tenuto*), the present article aims to assess both specific features of distinct touch and articulation strategies and the impact of trunk motion on these performance strategies. Two specific and related objectives are pursued: on the one hand, to estimate pianists’ upper-limb linear velocities when producing different types of keystrokes, and on the other hand, to effectively evaluate the contribution of the whole kinematic chain to the generation of endpoint linear velocities in terms of joint kinematics. Based on the approach to piano performance developed at Université de Montréal, we hypothesize that not only touch and articulation but also trunk motion might modify upper-limb segmental velocities and joint angular contribution to these velocities. In other words, we anticipate that trunk kinematics should also be considered as a relevant feature when evaluating and developing strategies aiming both to enhance motion efficiency and to reduce the risk of pianists’ PRMD.

## Materials and Methods

### Participants

Twelve expert pianists holding or currently pursuing a doctoral degree in piano performance at Université de Montréal volunteered to participate in the study. Only data of 9 pianists (2 females and 7 males; 34 ± 4.4 years old) were included because of recurrent occlusion of finger markers caused by the piano itself during specific trials of three participants. Pianists were fully advised of the experimental content, and each of them provided written informed consent. The study was approved by the Université de Montréal Ethics Committee (No. 18-086-CPER-D).

### Experimental Design

**FIGURE 1:**
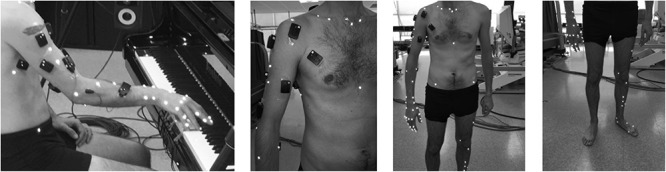
Location of markers for motion capture (participants also wore surface electromyography sensors, but these data are not discussed here).

Based on complementary kinematic models (e.g., Cerveri et al., 2007; Jackson et al., 2012), 68 reflective markers were placed on the following segments: pelvis, thorax, right upper limb, and left lower limb (Figure 1). The marker set included anatomical markers (located on bony landmarks for the model definition) and technical markers (located in areas that minimized both skin movement artifacts and marker occlusion for joint kinematics estimation during the task). First, pianists were asked to perform 2 static trials and a series of 9 setup movements. Then, they were asked to play repetitive keystrokes (A4) on a computer-controlled grand piano (Bösendorfer CEUS) at a high sound intensity level (*forte*, 82 dB) and a fixed slow tempo (30 bpm). Participants performed the keystrokes with the middle finger of the right hand to facilitate standardization and comparison between subjects. The tone target was previously recorded on the Bösendorfer piano and played to the participants by the piano’s reproducing system at the beginning of the experience. Sound intensity level was monitored to inform pianists if they differed more than ± 1 dB from the target tone. To ensure rhythm similarity across trials, tempo was repeatedly shown to participants with a metronome before the beginning of each condition.

Experimental conditions were determined by varying three independent variables: (i) body implication (use of trunk and upper-limb motion [trunk conditions] or use of only upper-limb motion [upper-limb conditions]); (ii) touch (pressed or struck), and (iii) articulation (*staccato* or *tenuto*). All participants performed in a randomized order the eight possible combinations of these three variables. The sustain pedal was held throughout each trial by the pianists’ right lower limb, and no indication was given related to either movement or position of the left lower limb. Two series of 20 keystrokes were performed for each condition. Data from the first and last keystrokes of each 20-tone trial were excluded from the analysis at a later stage (each condition accounted then for 36 keystrokes).

### Data Collection

Sound was recorded using a digital audio recorder (Sony PCM-D50) placed 1 meter away from the right side of the piano’s soundboard at a height of approximately 1.4 meters. A digital sound-level meter (Extech 407730), placed right beside the digital audio recorder, was used to obtain the actual sound pressure level (SPL) of the tone target. Pianists’ kinematics was collected using Nexus (version 2.6) and an 18 VICON camera motion analysis system (Oxford Metrics Ltd., Oxford, United Kingdom) at a sampling rate of 150 Hz. The lid of the grand piano was closed to reduce marker occlusion during the recording of trials.

### Data Processing

The sound level of keystrokes was estimated from the digital recording. Each series of A4 tones was segmented using MIRtoolbox for Matlab (Lartillot et al., 2008). The maximum magnitude value reached at the summit of the attack was detected for each tone, converted in dB, and shifted in order to match dB values obtained during the experiment (this was performed by adding to all dB values the distance between the mean dB value of all keystrokes and the actual SPL [82 dB] of the tone target obtained from the sound-level meter).

A static trial and setup movements (Begon et al., 2007; Michaud et al., 2016) acquired during the data collection were used to locate joint centers and to create a personalized 36-degree-of-freedom (DoF) kinematic model of each participant (pelvis, [root segment, 6 DoF; q_1__–__6_], thorax [q_7__–__9_], clavicle, scapula, and arm [3 DoF each; q_10__–__18_], forearm and wrist [2 DoF each; q_19__–__22_;], middle finger metacarpophalangeal joint [2 DoF; q_23__–__24_], thigh, shank, and foot [3 DoF each; q_25__–__33_], and head [q_34__–__36_]). Generalized coordinates (q) of the kinematic model for each experimental trial were reconstructed by solving an inverse kinematics problem using a weighted non-linear least-squares algorithm (Begon et al., 2008). To account for sporadic marker occlusion due to the fallboard of the grand piano, lower weightings (0.001 vs. 1) were given to the finger’s markers and only two DoF at the metacarpophalangeal joint were included in the model.

As the experimental task did not include hand mediolateral displacement on the keyboard, only vertical and anteroposterior velocities were computed for the 5 following points used to estimate endpoint and upper-limb linear velocities of joints.

– Fingertip: marker placed on the middle finger’s nail. This point was used as the reference of endpoint vertical velocity.
– Middle finger’s metacarpophalangeal joint (MCPJ) center. MCPJ served as the reference for endpoint anteroposterior velocity because the fingertip does not slide forward or backward on the key during the key descent in the context of the experimental task performed by pianists.
– Wrist, elbow, and glenohumeral (hereafter, shoulder) joint centers.

Five joints or groups of joints were defined to estimate joint contribution to endpoint velocity: pelvo-thoracic joints (including motion of the pelvis and of lumbar and thoracic spine), right shoulder-girdle joints, right elbow joint (including elbow flexion/extension and forearm pronation/supination), right wrist, and right middle finger’s metacarpophalangeal joint. When computed as the partial derivative with respect to the generalized coordinates, the endpoint velocity (*M*) can be expressed as the sum of the contributions of each joint of the kinematic chain (as in Begon et al., 2010):

(1) $$\mathrm{fingertip}\,\dot{M}=\underset{\mathrm{Pelvis}\mathrm{contr}.}{\underbrace{\frac{\partial M}{\partial q_{1-6}}\,{\dot{q}}_{1-6}}}+\underset{\mathrm{Thorax}\mathrm{contr}.}{\underbrace{\frac{\partial M}{\partial q_{7-9}}\,{\dot{q}}_{7-9}}}+\underset{\mathrm{Should}.\mathrm{girdle}\mathrm{contr}.}{\underbrace{\frac{\partial M}{\partial q_{10-18}}\,{\dot{q}}_{10-18}}}+\underset{\mathrm{Elbow}\mathrm{contr}.}{\underbrace{\frac{\partial M}{\partial q_{19-20}}\,{\dot{q}}_{19-20}}}+\underset{\mathrm{Wrist}\mathrm{contr}.}{\underbrace{\frac{\partial M}{\partial q_{21-22}}\,{\dot{q}}_{21-22}}}+\underset{\mathrm{MCPJ}\mathrm{contr}.}{\underbrace{\frac{\partial M}{\partial q_{23-24}}\,{\dot{q}}_{23-24}}}$$

(2) $$\mathrm{MCPJ}\,\dot{M}=\underset{\mathrm{Pelvis}\mathrm{contr}.}{\underbrace{\frac{\partial M}{\partial q_{1-6}}\,{\dot{q}}_{1-6}}}+\underset{\mathrm{Thorax}\mathrm{contr}.}{\underbrace{\frac{\partial M}{\partial q_{7-9}}\,{\dot{q}}_{7-9}}}+\underset{\mathrm{Should}.\mathrm{girdle}\mathrm{contr}.}{\underbrace{\frac{\partial M}{\partial q_{10-18}}\,{\dot{q}}_{10-18}}}+\underset{\mathrm{Elbow}\mathrm{contr}.}{\underbrace{\frac{\partial M}{\partial q_{19-20}}\,{\dot{q}}_{19-20}}}+\underset{\mathrm{Wrist}\mathrm{contr}.}{\underbrace{\frac{\partial M}{\partial q_{21-22}}\,{\dot{q}}_{21-22}}}$$

Linear velocities of the joints were computed using a three-point finite difference. Kinematic data was delimited by a window of 50 frames (333 ms) before and after the beginning of the finger–key descent (t_0_) for each keystroke. Instant t_0_ was estimated by comparing the fingertip’s vertical position in relation to a marker placed on the keyboard. Time differences between each t_0_ were adjusted for each trial by manually synchronizing segmentations of kinematic and sound data (segmentation of sound data was in fact a more robust procedure thanks to the abrupt and defined attack slope of loud piano tones). All keystrokes were divided into the same three phases to facilitate comparisons between the different experimental conditions.

– Attack-swing phase: starting at the beginning of the keystroke window (t_–__333__ms_).
– Attack phase: starting at t_0_.
– Follow-through phase: starting at t_40ms_ (the estimated latest key bottom time-point for all conditions). Due to sporadic fingertip real marker occlusion, the average time-point of the reconstructed fingertip minimum position after t_0_ across trials and participants was used to estimate the key bottom time-point. This time-point was calculated separately for struck and pressed conditions. Visual verification of the real and the reconstructed fingertip marker showed that the reconstructed downward motion of the fingertip slightly overextended for around 30 ms regardless of the type of touch (see Figure 3). This might be due to both the finger’s forward rotation over the key at the key bottom and the absence of the distal and middle finger phalanges in our kinematic model. The corrected key bottom time-points (after subtraction of the additional 30 ms) were then 27 ms after t_0_ for struck conditions and 40 ms after t_0_ for pressed conditions. These corrected values are consistent with the temporal description of loud (*forte*) pressed and struck tones reported in Goebl et al. (2005).

### Statistics

Statistical analysis was performed using the data of the 36 keystrokes of each subject per condition. In line with the three experimental independent variables (body implication, touch, and articulation), non-parametric three-way ANOVAs with repeated measurements were applied to both single-point and time-series values. Analysis of variance of time-series values was performed using the spm1d package (Pataky, 2010), which allows to perform statistical parametric mapping methods to time series (*p* < 0.05 and effect length > 20 ms were defined as thresholds to report significant differences of time-series values). When double and triple interactions were found, non-parametric one-way ANOVAs with repeated measurements were respectively applied to the group of conditions concerned.

Statistical parametric mapping of time-series values remains a more powerful tool to evaluate the impact of variables on the data collected (Robinson et al., 2015). Probability values tend to be higher when analyzing time-series histories and, therefore, risks of error are reduced. However, we decided to include statistical analysis of single-point values for two reasons. First is to perform statistical analysis on sound intensity (i.e., dB) values and on pianists’ kinematics at the theoretically estimated time-point when the hammer loses contact with the action mechanism to freely hit the strings. Since the pianist loses control over the hammer at this time-point, this moment is hereafter called the sound production time-point. According to Goebl et al. (2005), when producing loud tones on a grand piano, the sound production time-point occurs at virtually the same time that the key reaches the key-bottom. The sound production time-point was then defined at t_27 ms_ for struck conditions and at t_40 ms_ for pressed conditions (in line with the above-explained temporal differences between struck and pressed touch). Second, literature on the biomechanics of piano performance has been built on the analysis of single-point values. The performed statistical analysis on time-point values helps establish a better dialogue with findings of previous studies.

Three groups of single-point values were selected: SPL values as well as upper-limb joint linear velocities and joint contribution to endpoint velocities at the sound production time-point. The false discovery rate (FDR) procedure (Benjamini and Hochberg, 1995) was applied to the statistical analysis of single-point values to control for potential errors produced by multiple comparisons (*q* = 0.05; FDR = 5%). The effect size (Cohen’s d) was calculated for the analysis of variance of single-point values and was qualitatively interpreted as very large (*d* ≥ 1.2), large (1.2 > *d* ≥ 0.8), medium (0.8 > *d* ≥ 0.5), and small (0.5 > *d* ≥ 0.2) (Sawilowsky, 2009). Time-series values covered all three phases of the keystroke window and included (i) upper-limb joint linear velocities and (ii) joint contribution to endpoint velocities. Absolute difference and percentage difference of mean values are reported in some cases to better characterize significant differences found by the statistical analysis. Percentage difference was computed using as a reference the smallest of the two values being compared. In the case of time-series values, absolute difference and percentage difference were calculated for each point of the time-window where significant differences were found and only the mean absolute and percent differences are reported.

## Results

### Sound Intensity Levels

Sound pressure level values exhibited body implication, touch, and articulation main effects (*d* = 0.31 [small], *q* = 0.014; *d* = 0.34 [small], *q* = 0.014, and *d* = 0.22 [small], *q* = 0.014, respectively). Trunk conditions (mean ± standard deviation = 82.18 ± 0.92 dB) were louder than upper-limb conditions (81.81 ± 1.00 dB). Struck conditions (82.19 ± 1.03 dB) were louder compared to pressed conditions (81.80 ± 0.88 dB). Finally, *staccato* conditions (82.13 ± 0.86 dB) were louder than *tenuto* conditions (81.86 ± 1.07 dB). Absolute differences between mean dB values were however small: 0.37 dB (trunk vs. upper-limb conditions), 0.39 dB (struck vs. pressed conditions), and 0.27 dB (*staccato* vs. *tenuto* conditions).

### Upper-Limb Linear Velocities at the Sound Production Time-Point

**FIGURE 2:**
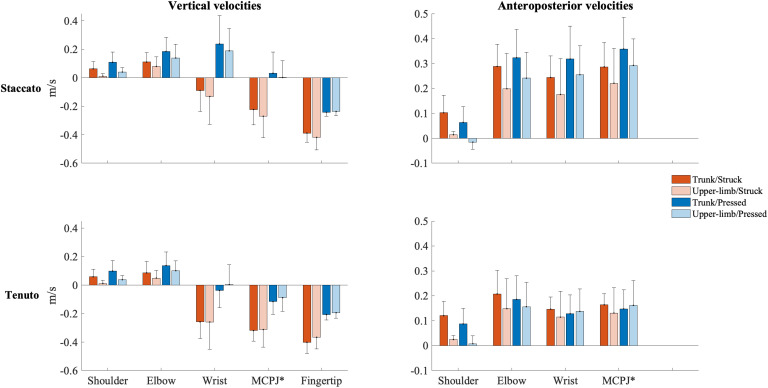
Mean upper-limb linear velocities at the sound production time-point. Error bars indicate ± 1 standard deviation. Negative/positive values indicate downward/upward velocities and backward/forward velocities. *Metacarpophalangeal joint.

Mean vertical velocity values at the sound production time-point showed a downward fingertip velocity and upward elbow and shoulder velocities (Figure 2). The direction of MCPJ and wrist vertical velocities depended on touch and articulation. Vertical velocities of all joints exhibited a touch effect, and the MCPJ and the wrist showed an articulation effect (Table 1). Struck conditions produced faster downward velocities (very large effects) than pressed conditions at the fingertip (absolute difference = 0.173 m/s, percentage difference = 79%) and the MCPJ (absolute difference = 0.238 m/s, percentage difference = 561%). At the wrist, struck conditions showed a higher downward velocity while pressed conditions presented either an upward velocity or a slower downward velocity (very large effect). Elbow upward velocity was faster in pressed than in struck conditions (large effect). Touch and body implication effects at the shoulder were uninterpretable due to a body implication/touch statistical interaction. All the respective groups of one-way ANOVA showed significant effects. On the one hand, trunk/pressed keystrokes created a faster shoulder upward velocity than trunk/struck keystrokes (*d* = 0.67 [medium], *q* = 0.011) while UL/pressed keystrokes produced a slower shoulder upward velocity than UL/struck keystrokes (*d* = 1.02 [large], *q* = 0.011). On the other hand, trunk keystrokes generated a faster shoulder upward velocity than UL conditions during both struck and pressed keystrokes (respectively, *d* = 1.22 [very large], *q* = 0.020; *d* = 1.14 [large], *q* = 0.014). Medium and large articulation effects occurred respectively on MCPJ and wrist vertical velocities: *staccato* conditions produced either upward velocities or slower downward velocities while *tenuto* conditions created greater downward velocities at these joints.

**TABLE 1:** Mean ± standard deviation values of upper-limb linear velocities (m/s) at the sound production time-point.

		**Vertical velocity**				**Anteroposterior velocity**			
		***Trunk***	***Upper-limb***	***d***	***q***	***Trunk***	***Upper-limb***	***d***	***q***
Body implication	*Fingertip*	−0.310 ± 0.121	−0.304 ± 0.136	–	0.775	–	–	–	–
	MCPJ*	−0.156 ± 0.177	−0.167 ± 0.186	–	0.775	0.239 ± 0.133	0.202 ± 0.131	–	0.246
	*Wrist*	−0.037 ± 0.239	−0.049 ± 0.245	–	0.775	0.209 ± 0.124	0.171 ± 0.127	–	0.183
	*Elbow*	0.130 ± 0.096	0.093 ± 0.081	–	0.143	0.251 ± 0.118	0.186 ± 0.122	0.54	**0.030**
	*Shoulder*	0.082 ± 0.068	0.025 ± 0.031	–	0.030**	0.093 ± 0.073	0.008 ± 0.031	1.52	**0.025**

		***Struck***	***Pressed***	***d***	***q***	***Struck***	***Pressed***	***d***	***q***

Touch	*Fingertip*	−0.394 ± 0.116	−0.220 ± 0.067	1.84	**0.011**	–	–	–	–
	MCPJ**	−0.281 ± 0.138	−0.043 ± 0.137	1.74	**0.011**	0.201 ± 0.121	0.240 ± 0.141	–	0.288
	*Wrist*	−0.184 ± 0.187	0.098 ± 0.205	1.44	**0.011**	0.170 ± 0.115	0.210 ± 0.135	–	0.267
	*Elbow*	0.082 ± 0.073	0.141 ± 0.097	0.69	**0.030**	0.211 ± 0.125	0.227 ± 0.123	–	0.681
	*Shoulder*	0.036 ± 0.047	0.072 ± 0.065	–	0.014**	0.065 ± 0.069	0.036 ± 0.069	–	0.107

		***Staccato***	***Tenuto***	***d***	***q***	***Staccato***	***Tenuto***	***d***	***q***

Articulation	*Fingertip*	−0.321 ± 0.125	−0.292 ± 0.130	–	0.141	–	–	–	–
	MCPJ*	−0.115 ± 0.194	−0.209 ± 0.155	0.53	**0.014**	0.290 ± 0.132	0.151 ± 0.091	1.22	**0.011**
	*Wrist*	0.052 ± 0.246	−0.138 ± 0.197	0.85	**0.011**	0.248 ± 0.133	0.132 ± 0.088	1.04	**0.011**
	*Elbow*	0.129 ± 0.093	0.094 ± 0.085	–	0.141	0.263 ± 0.124	0.174 ± 0.107	0.76	**0.030**
	*Shoulder*	0.056 ± 0.060	0.052 ± 0.059	–	0.696	0.041 ± 0.071	0.060 ± 0.069	–	0.080

p-Value corrected (*q*-value) from the three-way repeated-measures ANOVAs and effect size (Cohen’s d). Significance was set at *p* ≤ 0.05 and corrected with the false discovery rate procedure for multiple comparisons (*q* ≤ 0.05). Negative/positive values indicate downward/upward velocities and backward/forward velocities; *q*-values are in bold when significant. * Metacarpophalangeal joint. ** Uninterpretable effect due to a body implication/touch interaction.

Mean anteroposterior velocity values of all upper-limb joints showed a forward direction at the sound production time-point (Figure 2). The unique relevant exception to this pattern occurred at the shoulder during the upper-limb/pressed/*staccato* condition. All joints were affected by at least one main effect (Table 1). Compared to upper-limb conditions, trunk conditions created a faster forward velocity at the elbow (medium effect, absolute difference = 0.065 m/s, percentage difference = 35%) and the shoulder (very large effect, absolute difference = 0.086 m/s, percentage difference = 1094%). MCPJ, wrist, and elbow exhibited an articulation main effect. Forward velocities were respectively 92% (very large effect), 89% (large effect), and 51% (medium effect) higher in *staccato* conditions compared to *tenuto* conditions. No effect of touch was reported on anteroposterior velocity values.

### Upper-Limb Linear Velocities During the Whole Keystroke

#### Vertical Velocities

**FIGURE 3:**
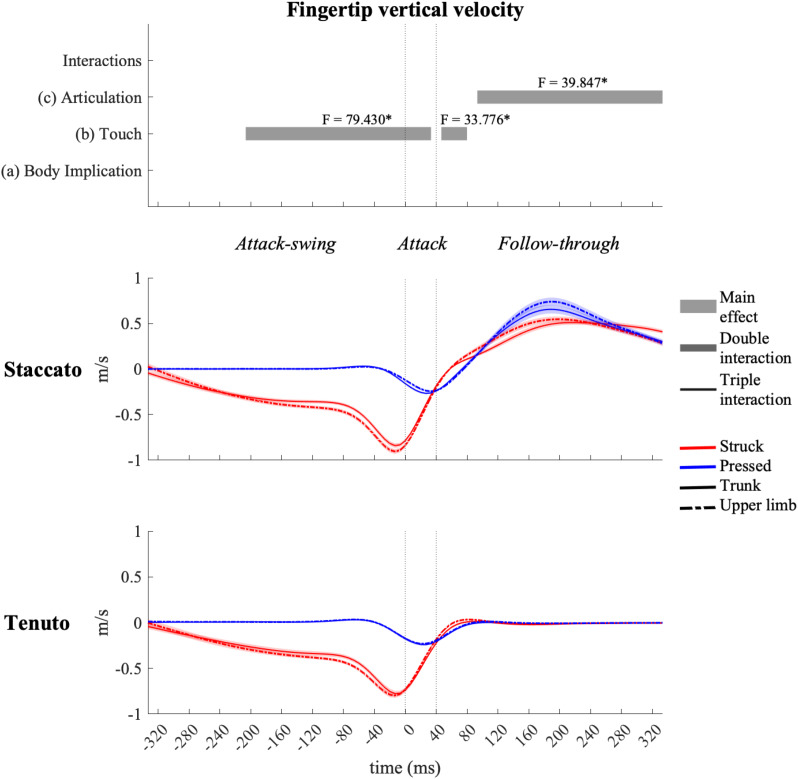
Mean values (solid and dash-dotted lines) and 95% bootstrap confidence interval (shaded areas) of the fingertip vertical velocity during the three phases of the keystroke. Negative/positive values indicate downward/upward velocities. The top panel represents the results from the statistical parametric mapping of time-series values. *p-value ≤ 0.001. As explained in the methods (Section “Data Processing”), the fingertip shows an overextended downward velocity for approximately 30 ms after reaching the end of the finger–key descent due probably to both the finger’s forward rotation over the key and the absence of the distal and middle finger phalanges in our kinematic model.

The fingertip exhibited the highest vertical velocity values in all types of keystrokes. As expected, statistical analysis showed an effect of touch and articulation on fingertip vertical velocity profiles (Figure 3). Compared to pressed touch, struck touch exhibited both a significantly higher fingertip downward velocity during the attack-swing and attack phases (absolute difference = 0.439 m/s, percentage difference = 3093%) and an earlier transition to an upward velocity at the very beginning of the follow-through phase. *Staccato* conditions created a fingertip upward velocity during almost all the follow-through phase while tenuto conditions produced a practically null fingertip vertical velocity (absolute difference = 0.467 m/s, percentage difference = 7410%).

**FIGURE 4:**
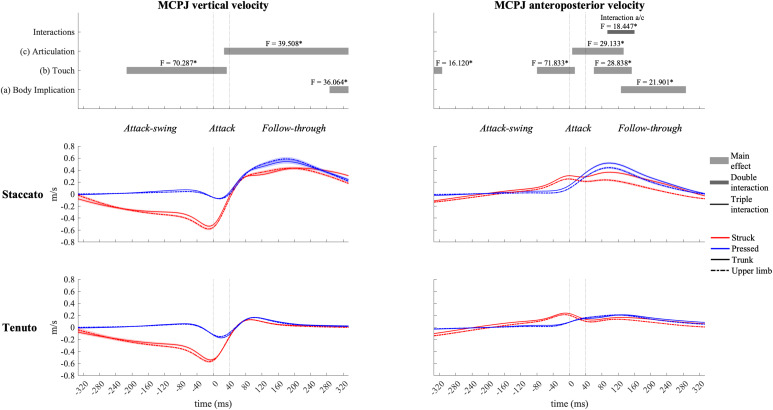
Mean values (solid and dash-dotted lines) and 95% bootstrap confidence interval (shaded areas) of metacarpophalangeal joint (MCPJ) linear velocities during the three phases of the keystroke. Negative/positive values indicate downward/upward velocities and backward/forward velocities. Top panels represent the results from the statistical parametric mapping of time-series values. *p-value ≤ 0.001.

**FIGURE 5:**
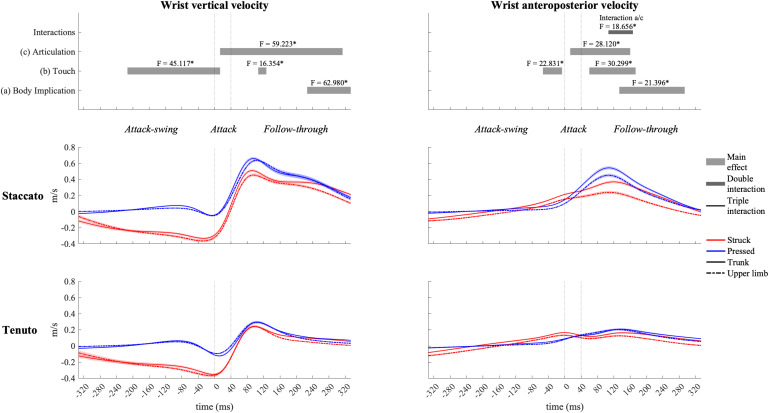
Mean values (solid and dash-dotted lines) and 95% bootstrap confidence interval (shaded areas) of wrist linear velocities during the three phases of the keystroke. Negative/positive values indicate downward/upward velocities and backward/forward velocities. Top panels represent the results from the statistical parametric mapping of time-series values. *p-value ≤ 0.001.

Vertical velocity profiles of the MCPJ (Figure 4) and the wrist (Figure 5) exhibited similar shapes and main effects compared to the fingertip: (i) struck conditions showed higher downward velocities than pressed conditions during the attack-swing and attack phases, and (ii) *staccato* conditions produced faster upward velocities than *tenuto* conditions during the follow-through phase. Despite these similarities with fingertip velocity, there were relevant differences. First, pressed conditions exhibited MCPJ and wrist upward velocities during the attack-swing phase. Second, there were articulation-related significant differences during the attack phase (particularly at the wrist, where an upward velocity was reported in pressed/*staccato* keystrokes). Third, not only *staccato* but also *tenuto* conditions exhibited upward MCPJ and wrist velocities during the follow-through phase. Four, there was a body implication main effect toward the end of the follow-through phase, where trunk conditions produced faster MCPJ and wrist upward velocities than upper-limb conditions.

**FIGURE 6:**
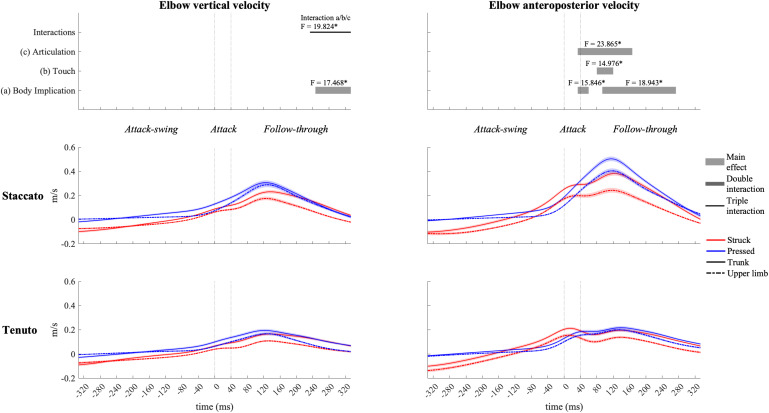
Mean values (solid and dash-dotted lines) and 95% bootstrap confidence interval (shaded areas) of elbow linear velocities during the three phases of the keystroke. Negative/positive values indicate downward/upward velocities and backward/forward velocities. Top panels represent the results from the statistical parametric mapping of time-series values. *p-value ≤ 0.001.

**FIGURE 7:**
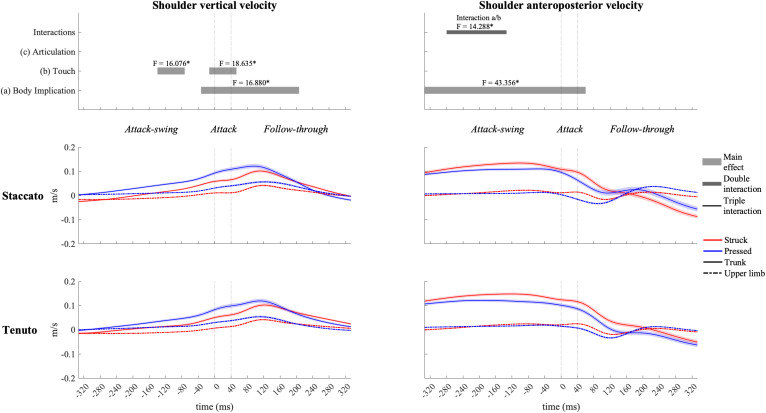
Mean values (solid and dash-dotted lines) and 95% bootstrap confidence interval (shaded areas) of shoulder linear velocities during the three phases of the keystroke. Negative/positive values indicate downward/upward velocities and backward/forward velocities. Top panels represent the results from the statistical parametric mapping of time-series values. *p-value ≤ 0.001.

The elbow (Figure 6) and the shoulder (Figure 7) displayed distinct vertical velocity profiles. Neither the elbow nor the shoulder exhibited an increase in their downward velocity approaching the attack phase. On the contrary, downward velocities decreased and upward velocities increased toward and during the attack phase. Peaks of elbow and shoulder upward velocities for all conditions were located in the first half of the follow-through phase. A triple interaction occurred in elbow vertical velocity at the end of the follow-through phase. During the triple interaction period, only a body implication effect was reported in specific groups of conditions. Trunk conditions exhibited a significantly faster elbow upward velocity than upper-limb conditions in struck/*staccato*, struck/*tenuto*, and pressed/*tenuto* keystrokes but not in pressed/*staccato* keystrokes (only struck/*staccato* keystrokes: *F* = 12.424, *p* = 0.005; only struck/*tenuto* keystrokes: *F* = 15.879, *p* = 0.005; only pressed/*tenuto* keystrokes: *F* = 13.536, *p* = 0.010). The vertical velocity of the shoulder showed a body implication effect. Trunk conditions presented a faster shoulder upward velocity than did upper-limb conditions from the end of the attack-swing phase to the second half of the follow-through phase (absolute difference = 0.030 m/s, percentage difference = 185%). There was also a touch effect in shoulder vertical velocity: pressed conditions displayed a higher upward velocity than struck conditions during both the attack-swing and attack phases.

#### Anteroposterior Velocities

All joints exhibited clear forward velocities for the most part of the keystroke window. MCPJ (Figure 4) and wrist (Figure 5) showed similar profiles and main effects (as in the case of their vertical velocity profiles). Before and during the attack, struck conditions created faster MCPJ and wrist forward velocities than pressed conditions (this touch effect was particularly relevant at the MCPJ [absolute difference = 0.130 m/s, percentage difference = 281%]). Right after the attack phase, pressed conditions created higher forward velocities. During distinct periods of the attack and follow-through phases, there were articulation and body implication effects: both *staccato* and trunk conditions produced faster MCPJ and wrist forward velocities compared respectively to *tenuto* and upper-limb conditions. A body implication/articulation interaction occurred during the follow-through phase (t_93__/__160_ [MCPJ] and t_107__/__167_ [wrist]). Two general results were extracted from the group of one-way ANOVA performed in this specific interaction window. First, *staccato* conditions created faster MCPJ and wrist forward velocities than *tenuto* conditions in both trunk and upper-limb keystrokes (only trunk keystrokes: [MCPJ] *F* = 37.826, *p* = 0.001; [wrist] *F* = 43.043, *p* = 0.001; only upper-limb keystrokes: [MCPJ] *F* = 10.390, *p* = 0.026; [wrist] *F* = 11.406, *p* = 0.011). Second, trunk conditions showed significantly higher MCPJ and wrist forward velocities than upper-limb conditions in *staccato* but not *tenuto* keystrokes (only *staccato* keystrokes: [MCPJ] *F* = 19.502, *p* = 0.001; [wrist] *F* = 19.998, *p* = 0.003).

The elbow (Figure 6) exhibited rather similar anteroposterior velocity profiles to those of the MCPJ and the wrist. It also showed body implication, touch, and articulation effects, but there were no double or triple interactions. Both trunk and *staccato* conditions produced a faster elbow forward velocity compared respectively to upper-limb and *tenuto* conditions during the end of the attack phase and a great part of the follow-through phase. A touch effect also occurred during the follow-through phase, where pressed conditions produced a higher elbow forward velocity compared to struck conditions.

The shoulder (Figure 7) exhibited distinct anteroposterior velocity profiles in comparison to the rest of the upper-limb joints, and it was heavily influenced by body implication effect. Trunk conditions showed a significantly higher shoulder forward velocity than did upper-limb conditions from the attack-swing to the beginning of the follow-through phase (absolute difference = 0.103 m/s, percentage difference = 1466%). A body implication/touch interaction occurred during the attack-swing phase. During this interaction window, significant differences between trunk and upper-limb conditions remained in both struck and pressed keystrokes (respectively, *F* = 64.575, *p* = 0.001; and *F* = 43.727, *p* = 0.001). In addition, a touch main effect was reported in trunk conditions for a short period of the attack-swing phase, where trunk/struck conditions created a significant faster shoulder forward velocity than did trunk/pressed conditions (t_–__160__/__–__133_, *F* = 8.854, *p* = 0.040).

### Joint Angular Contribution to Endpoint Vertical and Anteroposterior Velocities at the Sound Production Time-Point

Joints can be divided in two clearly distinct groups based on their angular contribution to fingertip vertical velocity at the sound production time-point (Table 2): on the one hand, a distal joint complex comprising the elbow, wrist, and metacarpophalangeal joint, which created a downward fingertip velocity (except for the metacarpophalangeal joint in *tenuto* conditions), and on the other hand, a proximal joint complex comprising the trunk and shoulder-girdle joints, which contributed mostly to upward fingertip velocity (i.e., to the opposite direction of the fingertip velocity during the keystroke). Elbow contribution showed a large touch effect. Its contribution to endpoint downward velocity was 85% higher in struck than in pressed conditions (absolute difference = 0.228 m/s). Contributions to endpoint vertical velocity of shoulder-girdle joints and wrist showed large articulation effects. Compared to *tenuto* conditions, *staccato* conditions produced both a higher shoulder-girdle contribution to fingertip upward velocity (absolute difference = 0.207 m/s, percentage difference = 206%) and a higher wrist contribution to fingertip downward velocity (absolute difference = 0.246 m/s, percentage difference = 427%).

**TABLE 2:** Mean ± standard deviation values of joint angular contribution to endpoint velocities (m/s) at the sound production time-point.

		**Contribution to fingertip vertical velocity**				Contribution to MCPJ* anteroposterior velocity			
		***Trunk***	***Upper-limb***	***d***	***q***	***Trunk***	***Upper-limb***	***d***	***q***
Body implication	*Pelvo-thoracic joints*	0.109 ± 0.116	0.022 ± 0.030	–	0.080	0.115 ± 0.085	−0.002 ± 0.019	1.90	**0.011**
	*Shoulder-girdle joints*	0.134 ± 0.217	0.274 ± 0.263	–	0.141	0.066 ± 0.137	0.140 ± 0.128	–	0.143
	*Elbow joints*	−0.347 ± 0.276	−0.410 ± 0.301	–	0.349	0.033 ± 0.034	0.038 ± 0.042	–	0.550
	*Wrist*	−0.180 ± 0.277	−0.182 ± 0.294	–	0.968	0.026 ± 0.043	0.025 ± 0.050	–	0.942
	MCPJ*	−0.025 ± 0.093	−0.007 ± 0.100	–	0.267	–	–	–	–

		***Struck***	***Pressed***	***d***	***q***	***Struck***	***Pressed***	***d***	***q***

Touch	*Pelvo-thoracic joints*	0.052 ± 0.081	0.078 ± 0.106	–	0.143	0.062 ± 0.082	0.051 ± 0.087	–	0.379
	*Shoulder-girdle joints*	0.136 ± 0.213	0.272 ± 0.267	–	0.080	0.074 ± 0.118	0.131 ± 0.149	–	0.143
	*Elbow joints*	−0.493 ± 0.264	−0.265 ± 0.271	0.85	**0.014**	0.046 ± 0.036	0.025 ± 0.037	–	0.253
	*Wrist*	−0.086 ± 0.256	−0.276 ± 0.282	–	0.143	0.018 ± 0.047	0.033 ± 0.046	–	0.567
	MCPJ*	−0.004 ± 0.072	−0.029 ± 0.115	–	0.567	–	–	–	–

		***Staccato***	***Tenuto***	***d***	***q***	***Staccato***	***Tenuto***	***d***	***q***

Articulation	*Pelvo-thoracic joints*	0.079 ± 0.098	0.051 ± 0.091	–	0.080	0.052 ± 0.090	0.061 ± 0.079	–	0.494
	*Shoulder-girdle joints*	0.308 ± 0.270	0.100 ± 0.178	0.91	**0.011**	0.162 ± 0.143	0.044 ± 0.103	0.95	**0.011**
	*Elbow joints*	−0.370 ± 0.299	−0.388 ± 0.281	–	0.697	0.036 ± 0.039	0.035 ± 0.037	–	0.941
	*Wrist*	−0.304 ± 0.277	−0.058 ± 0.236	0.96	**0.014**	0.040 ± 0.045	0.011 ± 0.044	0.65	**0.011**
	MCPJ*	−0.035 ± 0.116	0.002 ± 0.068	–	0.732	–	–	–	–

p-Value corrected (*q*-value) from the three-way repeated-measures ANOVAs and effect size (Cohen’s d). Significance was set at *P* ≤ 0.05 and corrected with the false discovery rate procedure for multiple comparisons (*q* ≤ 0.05). Negative/positive values indicate contributions to downward/upward finger velocity and to backward/forward hand velocity; *q*-values are in bold when significant. * Metacarpophalangeal joint.

Mean contribution values of all joints to endpoint anteroposterior velocity showed a forward direction [except pelvo-thoracic joints contribution in upper-limb conditions (Table 2)]. A very large body implication effect occurred at pelvo-thoracic joints. Their contribution to MCPJ forward velocity was 6242% higher in trunk than in upper-limb conditions (absolute difference = 0.117 m/s [trunk conditions = 0.115 ± 0.081 m/s, upper-limb conditions = −0.002 ± 0.016 m/s]). As in the case of endpoint vertical velocity, contribution of shoulder-girdle joints and wrist to MCPJ forward velocity was influenced by articulation: *staccato* conditions produced a higher contribution of these joints to MCPJ forward velocity than tenuto conditions (shoulder girdle: large effect, absolute difference = 0.118 m/s, percentage difference = 269%; wrist: medium effect, absolute difference = 0.029 m/s, percentage difference = 263%).

### Joint Angular Contribution to Endpoint Vertical and Anteroposterior Velocities During the Whole Keystroke

#### Pelvic and Thoracic Joint Contribution

**FIGURE 8:**
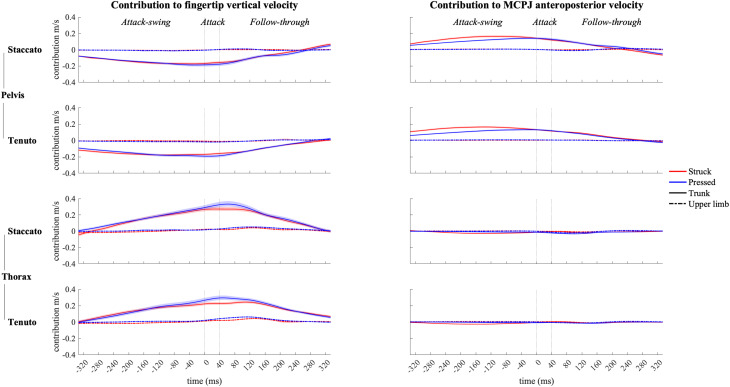
Mean values (solid and dash-dotted lines) and 95% bootstrap confidence interval (shaded areas) of pelvic joint and thoracic joint angular contribution to endpoint velocities during the three phases of the keystroke. Negative/positive values indicate contributions to downward/upward fingertip velocities and to backward/forward metacarpophalangeal joint (MCPJ) velocities.

Contribution profiles of pelvo-thoracic joints were highly influenced by body implication, upper-limb conditions presenting very small contribution values (Figure 8) since during the upper-limb conditions pianists were not supposed to actively use pelvo-thoracic joint motion to perform the keystrokes. When mobilized, pelvic joints contributed to downward and forward endpoint velocities from the beginning of the attack-swing phase to the middle of the follow-though phase. Thoracic joints contributed to an upward fingertip velocity during the whole keystroke window and showed a practically null contribution to MCPJ forward velocity. In upper-limb conditions, thoracic joints exhibited a noticeable but limited contribution to fingertip upward velocity in the follow-through phase, particularly in *tenuto* conditions.

**FIGURE 9:**
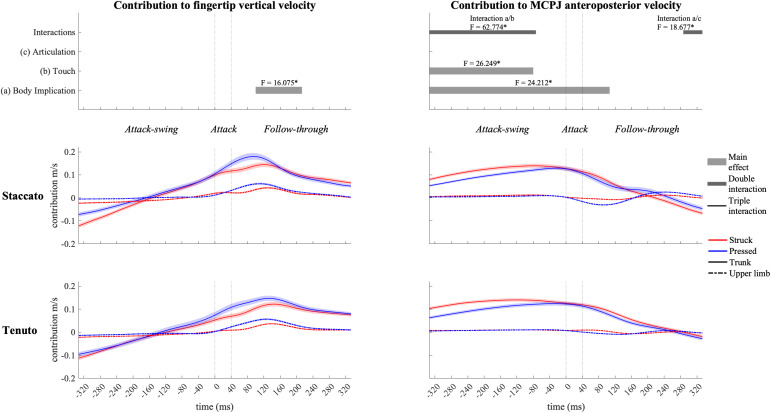
Mean values (solid and dash-dotted lines) and 95% bootstrap confidence interval (shaded areas) of pelvo-thoracic joint angular contribution to endpoint velocities during the three phases of the keystroke. Negative/positive values indicate contributions to downward/upward fingertip velocities and to backward/forward metacarpophalangeal joint (MCPJ) velocities. Top panels represent the results from the statistical parametric mapping of time-series values. *p-value ≤ 0.001.

When grouped together, pelvo-thoracic joints (Figure 9) showed significant differences in all three phases of the keystroke. Compared to upper-limb conditions, trunk conditions showed a significantly greater pelvo-thoracic joint contribution to endpoint upward velocity (follow-through phase) and to endpoint forward velocity (attack-swing, attack, and follow-through phases [absolute difference = 0.105 m/s, percentage difference = 1082%]). A body implication/touch interaction occurred in pelvo-thoracic joint contribution to MCPJ anteroposterior velocity during a great section of the attack-swing phase. During this interaction window, pelvo-thoracic joint contribution to MCPJ anteroposterior velocity remained significantly higher in trunk than in upper-limb conditions regardless of the type of touch (only struck keystrokes: *F* = 34.303, *p* = 0.001; only pressed keystrokes: *F* = 20.837, *p* = 0.001). There was also a touch effect in trunk but not in upper-limb conditions: trunk/struck keystrokes produced a higher contribution of pelvo-thoracic joints to MCPJ forward velocity than trunk/pressed keystrokes during the interaction period (*F* = 58.682, *p* = 0.001). At the end of the follow-through phase, there was a body implication/articulation interaction, where trunk/*staccato* conditions produced a greater pelvo-thoracic joint contribution to MCPJ backward velocity than trunk/*tenuto* conditions (*F* = 11.170, *p* = 0.013).

#### Shoulder-Girdle Joint Contribution

**FIGURE 10:**
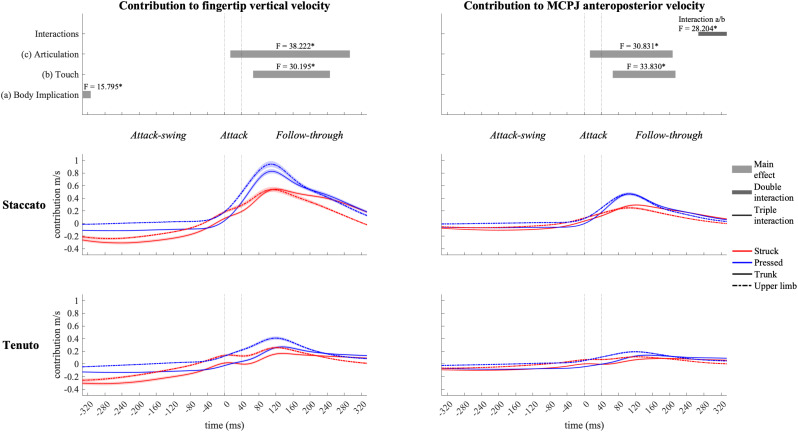
Mean values (solid and dash-dotted lines) and 95% bootstrap confidence interval (shaded areas) of shoulder-girdle-joint angular contribution to endpoint velocities during the three phases of the keystroke. Negative/positive values indicate contributions to downward/upward fingertip velocities and to backward/forward metacarpophalangeal joint (MCPJ) velocities. Top panels represent the results from the statistical parametric mapping of time-series values. *p-value ≤ 0.001.

Shoulder-girdle joints (Figure 10) contributed mainly to downward fingertip velocity and to limited but noticeable backward MCPJ velocity during the attack-swing phase (at the beginning of the keystroke window, shoulder-girdle contribution to endpoint downward velocity was higher in trunk than in upper-limb conditions). Approaching the attack and follow-through phases, shoulder-girdle joints contributed to endpoint upward and forward velocities. Articulation strongly affected shoulder-girdle-joint contribution during the attack and follow-through phases: *staccato* conditions created a higher contribution to upward (absolute difference = 0.297 m/s, percentage difference = 180%) and forward (absolute difference = 0.170 m/s, percentage difference = 207%) endpoint velocities than *tenuto* conditions. Shoulder-girdle contribution to endpoint vertical and anteroposterior velocities was also affected by touch during the follow-through phase: compared to struck conditions, pressed conditions generated a greater contribution to endpoint upward (absolute difference = 0.064 m/s, percentage difference = 35%) and forward (absolute difference = 0.092 m/s, percentage difference = 36%) velocities. A body implication/touch interaction in shoulder-girdle contribution to MCPJ anteroposterior velocity occurred at the end of the keystroke window. As contribution values were limited or practically null, no further statistical analysis was performed during this double interaction window.

#### Elbow Joint Contribution

**FIGURE 11:**
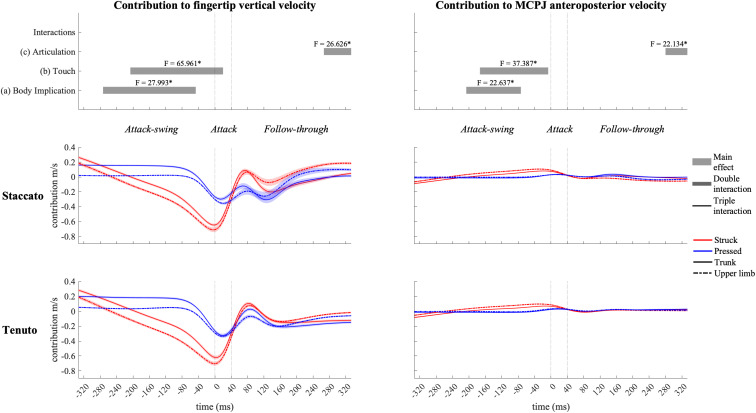
Mean values (solid and dash-dotted lines) and 95% bootstrap confidence interval (shaded areas) of elbow-joint angular contribution to endpoint velocities during the three phases of the keystroke. Negative/positive values indicate contributions to downward/upward fingertip velocities and to backward/forward metacarpophalangeal joint (MCPJ) velocities. Top panels represent the results from the statistical parametric mapping of time-series values. *p-value ≤ 0.001.

Elbow joint (i.e., elbow flexion and forearm pro-supination) contribution (Figure 11) had a prominent role in the creation of fingertip downward velocity while producing the attack. During the attack-swing and attack phases, struck conditions exhibited significantly greater elbow contribution to endpoint downward and forward velocities than pressed conditions. There was also a body implication effect during the attack-swing phase. Compared to upper-limb conditions, trunk conditions displayed (i) either a higher contribution to fingertip upward velocity or a lower contribution to fingertip downward velocity or (ii) a smaller contribution to MCPJ forward velocity (this significant difference is only relevant for struck conditions since elbow joints contribution to endpoint anteroposterior velocity in pressed conditions was practically null). An articulation main effect occurred at the very end of the follow-though phase. *Tenuto* conditions generated an elbow contribution to fingertip downward velocity while *staccato* conditions created an elbow contribution to fingertip upward velocity. The articulation effect in elbow contribution to MCPJ anteroposterior velocity was considered irrelevant as contribution values were rather limited.

#### Wrist Contribution

**FIGURE 12:**
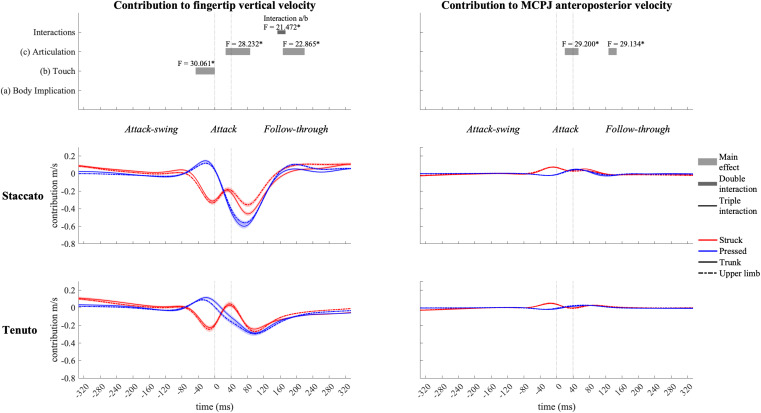
Mean values (solid and dash-dotted lines) and 95% bootstrap confidence interval (shaded areas) of wrist angular contribution to endpoint velocities during the three phases of the keystroke. Negative/positive values indicate contributions to downward/upward fingertip velocities and to backward/forward metacarpophalangeal joint (MCPJ) velocities. Top panels represent the results from the statistical parametric mapping of time-series values. *p-value ≤ 0.001.

Wrist angular contribution (Figure 12) was influenced by touch and articulation effects. Right before the beginning of the attack phase, the wrist joint contributed to a downward endpoint velocity in struck keystrokes and to an upward endpoint velocity in pressed keystrokes. An articulation effect occurred during the attack and follow-through phases in wrist contribution to both vertical and anteroposterior endpoint velocities. During the attack phase and the beginning of the follow-through phase, *staccato* conditions exhibited a higher wrist contribution to endpoint downward and forward velocities than *tenuto* conditions. In the middle section of the follow-through phase, the wrist contributed to fingertip upward velocity in *staccato* conditions and to fingertip downward velocity in *tenuto* conditions. A simultaneous articulation effect in wrist contribution to endpoint anteroposterior velocity was considered irrelevant as contribution values were practically null. During a body implication/touch interaction in the follow-through phase (from t_153_ to t_173_), the wrist generated a greater fingertip upward velocity in upper-limb/struck than in trunk/struck keystrokes (*F* = 12.928, *p* = 0.007).

#### Metacarpophalangeal Joint Contribution

**FIGURE 13:**
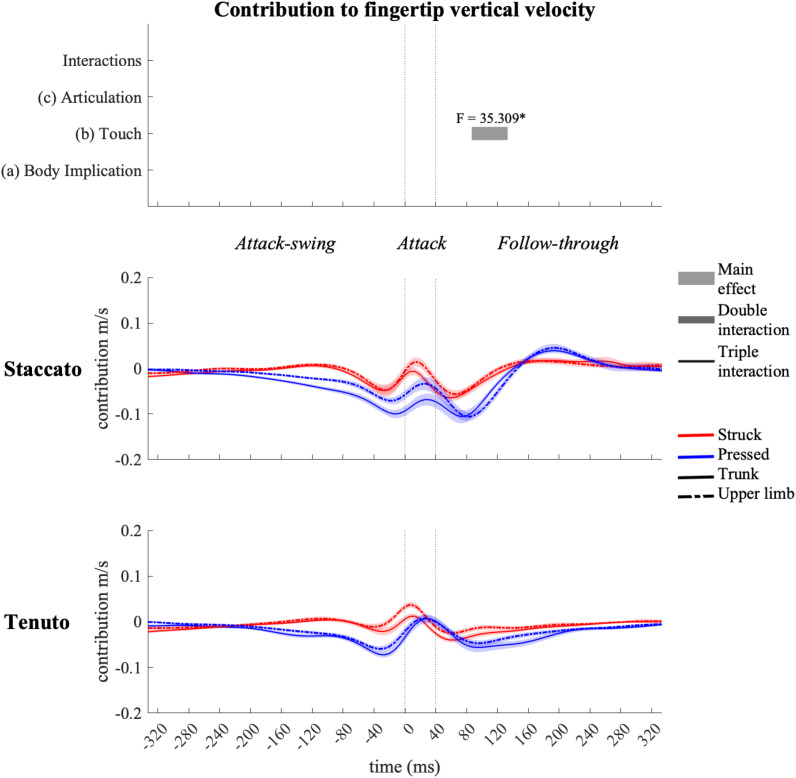
Mean values (solid and dash-dotted lines) and 95% bootstrap confidence interval (shaded areas) of metacarpophalangeal joint angular contribution to endpoint vertical velocity during the three phases of the keystroke. Negative/positive values indicate contributions to downward/upward fingertip velocities. Top panels represent the results from the statistical parametric mapping of time-series values. *p-value ≤ 0.001.

Metacarpophalangeal joint contribution (Figure 13) to fingertip vertical velocity was limited compared to other joints. There was only a touch effect during a short period of the follow-through phase, where metacarpophalangeal joint contribution to fingertip downward velocity was higher in pressed than in struck conditions.

## Discussion

This study is the first attempt to evaluate upper-limb joint linear velocities while integrating the motion of the pelvis and the thorax. It also represents the first research assessing joint angular contribution not only to vertical but also to anteroposterior endpoint velocities in the context of piano performance. Building on literature focused on features of different types of touch, the experimental design included pressed and struck touch, *staccato* and *tenuto* articulation, and two types of body implication strategies (i.e., trunk vs. upper-limb conditions). Our analysis highlighted that not only touch but also both the choice of body implication strategy and the type of articulation might have an impact on parameters affecting pianists’ performance such as sound intensity levels, upper-limb joint linear velocities, and joint contribution to generate velocity at the very end of the kinematic chain.

### Sound Intensity Levels

Even though pianists were asked to play at the same sound intensity level, maximum dB values of trunk, struck, and *staccato* conditions were statistically higher compared to those of upper-limb, pressed, and *tenuto* conditions, respectively. Nevertheless, size effects were small and only minor differences in mean SPL values were reported (0.37 dB [trunk vs. upper-limb], 0.39 dB [struck vs. pressed], and 0.27 dB [*staccato* vs. *tenuto*]). The just-noticeable difference (JND) for loudness varies not only along the loudness continuum but also according to timber. Depending on the context, however, the JND is commonly included in a 0.5- to 3-dB range (Parker and Schneider, 1980; Allen and Neely, 1997). Therefore, the reported differences in mean sound intensity values were smaller than the commonly used JND for loudness. Studies have shown that average listeners can differentiate struck and pressed touch because of a finger–key noise produced by struck touch right before the piano tone is produced (Goebl et al., 2004). In addition, listeners perceive struck touch as louder due to that same finger–key noise (Furuya et al., 2010; Goebl et al., 2014). Our analysis on sound intensity was performed using only maximum dB values of each keystroke, and consequently, anticipatory noises (such as the finger–key noise) did not affect sound intensity values. Therefore, there is no reason to assume that the reported statistically significant but subtle differences in SPL values were perceived by pianists. Differences on SPL values might then be more related to distinct biomechanical features of trunk/upper-limb, of struck/pressed, and of *staccato*/*tenuto* conditions than to the pianists’ capacity to produce perceivable equivalent loud tones while using different types of touch, articulation, and body implication strategies. These distinct features will be addressed in the next sections.

### Struck vs. Pressed Touch

#### Upper-Limb Linear Velocities—Touch

Our results indicate that touch significantly affects upper-limb velocities before, during and after the attack. Upper-limb joints showed two clearly distinct touch-related linear velocity profiles. Struck touch was characterized by higher downward velocities before and during the attack phase as well as by higher forward velocities right before the attack. Pressed touch produced greater upward and forward velocities respectively before and after the attack. It seems then that pianists, in the case of pressed keystrokes, compensated the fingertip’s virtually null motion during the attack-swing phase by creating (i) upward velocities of proximal upper-limb segments to anticipate the attack and (ii) faster MCPJ, wrist, and elbow forward accelerations from the beginning of the attack phase (as indicated by the anteroposterior velocity slopes of their joint centers from time-point t_0_ to approximately t_160_ in Figures 4–6). These results suggest that struck touch and pressed touch imply two distinct multi-joint movement organizations not only during the attack phase, as shown by Furuya et al. (2010), but also during the attack-swing and follow-through phases. Roughly speaking, struck touch created a downward-forward upper-limb thrust while pressed touch produced an upward-forward upper-limb thrust. Three ideas can be extracted from these distinct touch-related upper-limb types of thrusts.

First, better control of the key-descent and sound (Goebl et al., 2005, 2014), improved timing accuracy (Goebl and Palmer, 2008), and reduction of distal segments load (Kinoshita et al., 2007; Furuya et al., 2010) are not the only parameters in play when it comes to the pianist’s choice of touch. Indeed, choice between struck and pressed touch might be dictated by the score itself, i.e., by the compatibility between (i) the kinematic features of these two types of touch before, during, and after the attack and (ii) the spatiotemporal demands of the musical excerpt to be performed. In this sense, strategies aiming to improve mechanical or physiological efficiency of both types of touch might represent more adapted and practical tools to help enhance pianists’ performance and reduce the risk of developing PRMD.

Second, forward-joint velocities produced by the upward-forward upper-limb thrust of pressed touch seem to be an important feature of this type of touch to compensate for slower fingertip downward velocity at the sound production time-point. According to Kinoshita et al. (2007), pianists modulate sound intensity by adjusting three interdependent elements: (i) segment velocities (louder tones imply higher velocities at more proximal upper-limb segments); (ii) the effective mass of the keystroke (i.e., not the pianists’ mass but the specific portion of the pianists’ mass involved in the keystroke); and (iii) joint stiffness (i.e., a certain degree of muscle co-activation at specific joints to support the keystroke impact and, therefore, to effectively apply the desired effective mass on the keys). Kinoshita et al. (2007) showed that, since pressed touch produces a considerably slower fingertip downward velocity during the attack phase, it relies on a greater effective mass than struck touch to produce similar loud tones. Our results are in line with these authors’ findings as struck touch produced substantially faster (very large effects) fingertip (79%) and MCPJ (561%) downward velocities than pressed touch at the sound production time-point. This means that pianists must have used a more important effective mass in pressed keystrokes to reach the tone loudness of the experimental task. However, joint linear velocity profiles show that the arm and the scapula were mobilized in an upward direction during the attack phase regardless of the type of touch. Therefore, effective application of the mass of proximal upper-limb segments cannot rely on downward-joint velocities but rather on forward-joint velocities. Consequently, in pressed-touch conditions, pianists probably used a greater upper-limb effective mass that was mobilized in a forward rather than in a downward direction. During the key descent, as the fingertip does not slide on the key, the finger acts as a pivot point for the forward motion of the hand (Furuya and Kinoshita, 2008). The kinetic energy of a greater effective mass mobilized in a forward direction might then be effectively used to push the key downward if an adequate level of joint stiffness (muscle co-activation) is created at the finger joints. This strategy might have helped pianists increase the finger downward push on the key in pressed touch to produce the required loudness level.

Third, our results suggest that pianists seem to have a better control of forward segmental velocities in pressed than in struck touch. Anteroposterior velocity slopes of the MCPJ, wrist, and elbow show that pressed touch produced a smoother increase and decrease in forward velocities than struck touch. In struck keystrokes, forward velocities of these joints decreased at the beginning of the attack and increased right after the attack. On the contrary, no attack-related decrease in forward velocities was documented in pressed touch. Movement smoothness represents an important factor for the minimization of movement error (Salmond et al., 2016). Therefore, our results indicate that the greater keystroke effective mass of pressed touch reported in Kinoshita et al. (2007) not only helps pianists reach a certain sound intensity level but also enhances control of upper-limb anteroposterior-joint/segment velocities during and after the attack. Touch-related differences in control of anteroposterior velocities might for instance help explain the reported statistically significant differences in sound intensity between struck and pressed touch. This remains however a hypothesis. Links between control of sound and control of upper-limb linear motion should be investigated to gain further knowledge on the biomechanical and acoustical features of these two types of touch.

#### Joint Angular Contribution to Endpoint Velocities—Touch

Elbow joints had the most prominent role in creating fingertip downward velocity. Contribution profiles of these joints were greatly affected by touch during the attack-swing and attack phases. Compared to pressed touch, struck touch produced an 85% higher elbow joint contribution to fingertip downward velocity at the sound production time-point (a large effect mainly induced by faster elbow extension velocities). These results are in line with those of Furuya et al. (2010), where a significantly greater elbow extension velocity was found in struck touch during the attack phase. Furuya et al. (2009) showed also that to produce loud tones with a struck touch, expert pianists reduce elbow’s anti-gravity muscular (i.e., biceps and brachialis) activity. Therefore, the reported greater elbow contributions to endpoint downward and forward velocities during struck touch might have been facilitated by non-muscular forces (i.e., gravity effects). Lower levels of muscle co-activation at the elbow in struck keystrokes probably played an important role in both creating greater fingertip downward velocities and decreasing the effective mass implicated in the attack in comparison with pressed keystrokes.

Wrist contribution profiles of struck and pressed keystrokes suggest that this joint was affected in a greater manner by the inertia of the key in struck keystrokes. While the wrist contributed to the fingertip’s downward swing right before the attack (wrist flexion), its contribution values decreased at the finger–key contact time (t_0_) and tended to increase again toward the end of the attack (t_40_). On the contrary, in pressed keystrokes, the wrist anticipated the attack by contributing to endpoint upward velocity (wrist extension) and steadily increased its contribution to endpoint downward velocity (wrist flexion) from t_0_ to t_80_, approximately. According to Oikawa et al. (2011), while performing isolated repetitive keystrokes, the wrist flexor acts as an agonist of the hand’s downward motion while the wrist extensor acts as a continuous stabilizer. Based on this suggestion, we theorize that pressed touch probably produced higher levels of muscle activity to stabilize the wrist, reducing the impact of the inertia of the key on wrist angular motion. Smoother slopes of wrist contribution to fingertip downward velocity in pressed touch during the attack might be linked to the smoother key velocity profiles of pressed touch compared to struck touch documented by Goebl et al. (2005).

Furuya et al. (2010) reported significantly faster shoulder and metacarpophalangeal joint-flexion velocities in pressed touch than in struck touch during the attack. These authors suggested that faster shoulder and metacarpophalangeal flexion velocities in pressed touch might help pianists to rapidly increase the attack angle at the finger–key interaction point, as an upstanding finger position produces a mechanically advantageous attack angle (Harding et al., 1993). Our results indicate that both struck and pressed touch produced a simultaneous contribution of shoulder-girdle joints to endpoint upward and forward velocities (related to shoulder flexion) and of the metacarpophalangeal joint to fingertip downward velocity (related to flexion of this joint) during the attack. However, contributions of these joints were significantly greater in pressed keystrokes only during the follow-through phase. These findings suggest us three ideas. First, the use of shoulder flexion velocity during the attack could be a kinematic feature equally important for both pressed and struck touch. Second, the production of simultaneously greater shoulder and metacarpophalangeal joint-flexion velocities might be a specific kinematic feature of pressed touch during the follow-through phase and not necessarily the attack phase. Third, shoulder-girdle joints play a prominent role in producing a greater endpoint forward velocity in pressed keystrokes during the follow-through phase.

### Staccato vs. Tenuto Articulation

#### Upper-Limb Linear Velocities—Articulation

The reported significantly higher fingertip upward velocity in *staccato* keystrokes during the follow-through phase was expected as *tenuto* articulation implies a practically null fingertip motion after the attack. Apart from this expected difference, at least two relevant articulation-related findings can be extracted from our results.

On the one hand, the reported articulation effect on joint velocities after the attack suggests that expert pianists use distinct follow-through strategies according to articulation. While *staccato* keystrokes produced significantly higher upward (wrist and MCPJ) and forward (elbow, wrist, and MCPJ) velocities during the follow-through phase, *tenuto* keystrokes displayed clear upward and forward velocities of these joints despite the absence of motion of the fingertip. In *tenuto* keystrokes, pianists might have used upward and forward segmental velocities as a strategy to absorb impact forces generated by the key/key-bottom impact [a strategy somehow similar to landing eccentric phase after a jump (Norcross et al., 2013)]. In *staccato* keystrokes, greater upward and forward fingertip and joint velocities associated with higher fingertip range of motion during the follow-through phase could probably help pianists avoid stress on joints that would be caused by abrupt velocity drops [like in the case of the follow-through of a tennis strike (Reid et al., 2013)].

On the other hand, the reported results show that the choice of articulation modulates fingertip and joint velocities not only after but also during the attack and, more importantly, at the sound production time-point. Upward (wrist and MCPJ) and forward (elbow and wrist) velocities were higher in *staccato* keystrokes during the attack phase. At the specific sound-production time-point, *tenuto* keystrokes produced faster wrist and MCPJ downward velocities and *staccato* keystrokes produced higher MCPJ (92%), wrist (89%), and elbow (50%) forward velocities. These findings suggest first that the statistically higher sound intensity values of staccato keystrokes relied on greater forward segmental velocities at the sound production time-point (which probably produced a greater forwardly mobilized keystroke effective mass). Second, our findings highlight the fact that as articulation affects joint velocities during the key descent, results from studies in biomechanics and motor control addressing only one type of articulation may not be directly generalized to other types of articulation.

#### Joint Angular Contribution to Endpoint Velocities—Articulation

As in the case of linear velocities, articulation affected joint angular contribution to endpoint velocities not only after but also during the attack. Starting at approximately 20 ms after the beginning of the attack, *staccato* keystrokes produced 180% and 207% higher shoulder-girdle-joint contribution to endpoint upward and forward velocities than *tenuto* keystrokes, respectively. This substantial articulation effect lasted for a great section of the follow-through phase. Our results suggest that the shoulder girdle (a proximal joint complex) is the most important group of joints in the creation of the greater upward and forward distal segmental velocities associated with *staccato* keystrokes.

Wrist also exhibited considerably higher contributions to endpoint velocities in *staccato* than in *tenuto* conditions during the attack and follow-through phases. The shoulder girdle and the wrist play then a key role in modulating the distinct attack and follow-through kinematic strategies of *staccato* and *tenuto* articulation. Articulation effects at these joints were generally large at the sound-production time-point: *staccato* conditions produced a higher wrist contribution to downward (427%) and forward (263%) endpoint velocities as well as a greater shoulder-girdle angular contribution to upward (206%) and forward (269%) endpoint velocities. Furuya et al. (2010) suggested that greater shoulder-flexion velocity in pressed touch during the attack phase is generated by an effective utilization of distal-to-proximal intersegmental dynamics. Specifically, the analysis of these authors, based only on *staccato* keystrokes, indicated that a greater shoulder flexion velocity found in pressed touch was induced by torques created at more distal joints (elbow and wrist). Our results in wrist and shoulder-girdle-joint contribution to endpoint velocities suggest however that torques produced at the shoulder and the wrist could be affected by different types of articulation in the context of pressed and struck touch. It is then not possible to assume that the results of Furuya et al. (2010) can be generalized to *tenuto* keystrokes or other types of articulation. First, as above-mentioned, we did not find touch-related significant differences in shoulder-girdle-joint contribution to endpoint velocities in the attack phase. This indicates that the higher shoulder flexion velocities in pressed keystrokes during the attack reported by these authors might have been a kinematic strategy specific to the group of pianists that participated to their study. Second, a higher shoulder flexion velocity induced by torques created at more distal joints might have been a distal-to-proximal motion organization of this group of pianists to perform not any type of pressed keystroke but a pressed/*staccato* keystroke specifically. In this sense, control of multi-joint movements during the attack might not depend only on touch but also on articulation.

### Trunk vs. Upper-Limb Body Implication Strategy

#### Upper-Limb Linear Velocities—Body Implication

Different studies have shown that combat sports athletes increase their inertial contribution to the momentum transfer during the impact of strikes by augmenting both segmental velocities and the effective mass involved in the strike (Neto et al., 2007; McGill et al., 2010; Lenetsky et al., 2015). Greater segmental velocities and effective mass of strikes have been reported in boxing-punching strategies that take advantage of lower-limb and trunk motion (Cabral et al., 2010; Cheraghi et al., 2014). Our results suggest that it is possible to apply similar strategies to piano performance, i.e., that pianists could use pelvis and thorax motion to increase both segmental velocities and the effective mass of the keystroke.

During the attack-swing and attack phases, choice of body implication strategy when producing similarly loud keystrokes had a substantial influence on more proximal segment/joint linear velocities and no impact on endpoint velocities. In comparison to upper-limb conditions, trunk conditions generated (i) higher shoulder upward and forward velocities during all the phases of the keystrokes and (ii) a greater elbow forward velocity during the attack and follow-through phases. At the specific sound production time-point, trunk conditions showed 1094% and 35% higher forward velocities, respectively, at the shoulder and the elbow (very large and medium effects). These results indicate that the statistically higher sound intensity values of trunk conditions probably relied on faster-forward velocities of heavier body segments. As the greater forward velocity of the shoulder in trunk conditions was the product of a coordinated motion of pelvic, thoracic, and shoulder-girdle joints, the potentially greater effective mass of keystrokes involving trunk motion might relate not only to upper-limb segments but also to trunk segments. Goebl et al. (2005) showed that struck touch has the potential to produce louder tones than pressed touch does. Based on our results, by using motion of pelvo-thoracic joints, pianists’ might enhance their potential to produce louder tones in the context of both pressed and struck touch.

Empirical studies (e.g., Furuya et al., 2009) and theoretical works (e.g., James, 2018) have highlighted the physiological and mechanical benefits of effectively using gravity during the upper-limb downswing to attack the keys. Our results indicate that at the sound-production time-point, only the mean vertical velocity of the fingertip exhibited a downward direction in all types of keystrokes, while practically all joint anteroposterior velocities displayed a forward direction, regardless of the type of touch and articulation. Enhancing efficiency of the upper-limb forward swing to attack the keys by using trunk motion might then be a strategy to reduce risks of pianists’ PRMD that could be adapted to a wide variety of keystrokes (particularly when producing louder tones such as *forte* and *fortissimo*). Different types of articulation and touch can however induce a different balance between vertical and anteroposterior segmental velocities when mobilizing pelvo-thoracic joints. The reported triple and double interactions in joint linear velocities at several moments of the keystroke indicate that both the magnitude and the direction of joint vertical and anteroposterior velocities might depend on multiple and complex relations between body implication strategy, touch, and articulation.

Body implication effects in joint forward velocities showed a proximal-to-distal temporal sequence. Compared to upper-limb conditions, forward velocities in trunk conditions were faster at the shoulder (− 333 ms to 60 ms), the elbow (33 ms to 60 ms and 93 ms to 273 ms), the wrist (166 ms to 293 ms), and the MCPJ (160 ms to 286 ms). These temporal differences suggest that in trunk conditions, pianists needed a longer time to decelerate a probably greater MCPJ and wrist forward linear momentum generated at more proximal body segments. Whole-body proximal-to-distal transfer of momentum has not been studied in piano performance [the literature has for instance addressed upper-limb proximal-to-distal sequence (Furuya and Kinoshita, 2007) and intersegmental dynamics (Furuya et al., 2010)]. Several studies have however documented the existence of proximal-to-distal transfer of momentum from the lower limbs and the trunk to the upper limbs in sports such as tennis (Wang et al., 2010), baseball (Kageyama et al., 2014), and team-handball throw (van den Tillaar and Ettema, 2009). Future research including collection and analysis of contact and reaction forces (at the keys, the bench, and the floor) would allow a better understanding of the role of the entire kinematic chain to the momentum transfer from the pianist’s body to the keys.

#### Joint Angular Contribution to Endpoint Velocities—Body Implication

Inter-subject variability of upper-limb and pelvo-thoracic joint contribution to endpoint velocities is important (see standard deviation in Table 2). Previous studies have shown the presence of substantial inter-subject variability in pianists’ upper-limb kinematics (Dalla Bella and Palmer, 2011; Van Vugt et al., 2013). Our study supports this by showing that inter-subject variability in pianists’ kinematics might occur not only at upper-limb joints but also at pelvo-thoracic joints. Participants of the present study were all former or current doctoral students of the piano department of Université de Montréal. Therefore, all the participants had a certain level of familiarity with the specific approach to piano performance taught at this educational institution. This is probably why despite the reported important levels of inter-subject variability, all the pianists performed similar types of pelvo-thoracic joint angular motion. In fact, across the whole keystroke window, inter-subject kinematic variability at pelvic and thoracic joints was more related to differences in timing and in magnitude of motion than to the direction of angular motions. Specifically, when the pelvis and the thorax were mobilized (trunk conditions), the pianists that participated to the study mainly used pelvic anterior rotation and spine extension during the attack-swing, attack, and follow-through phases. In addition, as it can be deducted from the contribution slopes shown in Figure 8, pianists anticipated this “forward thrust” of the trunk by a backward trunk motion, implying anticipatory pelvic posterior rotation and spine-flexion movements to produce the trunk forward thrust.

Trunk and lower-limb contributions to enhance performance by increasing endpoint force or velocity has been documented in sitting sports such as kayaking (Begon et al., 2010) and tennis wheelchair (Cavedon et al., 2014). The reported greater contribution of pelvo-thoracic joints to endpoint forward and upward velocities in trunk than in upper-limb conditions show that trunk motion might have significant impacts in the creation of distal velocities during piano performance (a “sitting” music performance discipline). On the one hand, while preparing and producing the attack, pelvic anterior rotation produced the greater contribution of pelvo-thoracic joints to MCPJ forward velocity. On the other hand, while releasing or holding the keys, both pelvic anterior rotation and thoracic spine extension were responsible for the higher contribution of pelvo-thoracic joints to endpoint forward and upward velocities, respectively (facilitating the production of a whole-body follow-through motion after the keystroke impact). The use of these specific movements of the pelvic and the thorax significantly modified contributions of elbow joints during a great section of the attack-swing phase. Specifically, by implicating pelvo-thoracic joint motion, pianists modified the anticipatory motion of elbow joints (a key joint group in the creation of fingertip downward velocity) in two distinct touch-dependent ways: in struck touch, they relied less on elbow extension to produce the fingertip and MCPJ downward-forward thrust; in pressed touch, pianists were able to use forearm supination and elbow flexion to perform an anticipatory upward MCPJ and wrist movement as a strategy probably aiming to compensate for the impossibility to generate a fingertip attack-swing motion before the beginning of the key descent. Null or limited body implication effects on contributions of the shoulder girdle, the wrist, and the metacarpophalangeal joint suggest that the pianists modulated the angular motion of these joints in different ways when implicating trunk motion in the keystroke.

The reported interactions in contributions of pelvo-thoracic joints show that trunk magnitude of motion in the sagittal plane increased when endpoint spatiotemporal constraints were less important. Indeed, trunk conditions showed greater pelvo-thoracic joint contributions to (i) MCPJ forward velocity in struck than in pressed keystrokes during the attack-swing phase and (ii) MCPJ backward velocity in *staccato* than in *tenuto* keystrokes at the end of the follow-through phase. As tempo highly affects finger and hand spatiotemporal constraints (Furuya et al., 2012), pianists’ use of trunk motion might differ when producing slower and faster keystrokes. An early study by Bernstein and Popova (see Kay et al., 2003) showed that if at a slow tempo the upper limb produced an isolated motion for each keystroke, pianists performed an “elastic oscillation” upper-limb movement at a faster tempo, where single slower movements encompassed several smaller and faster distal movements [some pianists use the notion of synthesized movements to describe this type of complex multi-joint motion (Fink, 1992)]. The same rational could be applied to pelvis and thorax motion. In an actual performance, pianists might use kinematic strategies where several faster keystrokes are encompassed by a single slower trunk thrust. This idea establishes a bridge with theoretical and empirical literature addressing links between pianists’ gestures and expressive intentions (Jensenius et al., 2010; Thompson and Luck, 2012; Massie-Laberge et al., 2019). In an actual performance context, pianists’ movement efficiency and proficiency might involve several interlocked macro and micro movements (Kim et al., 2010) of upper-limb and trunk segments which can simultaneously serve both actual production of sound and realization/communication of musical structure.

## Limitations

While this article contributes to the advancement of knowledge on the effect of trunk motion, touch, and articulation in pianists’ kinematics, some limitations could be addressed by future research. As there was no previous evidence on the role of trunk motion in the creation of upper-limb segment velocities, our experimental design focused on a very simple task (isolated loud and slow-paced keystrokes) to evacuate performance features that could potentially affect the studied dependent variables (e.g., mediolateral displacement on the keyboard, expressive content of actual excerpts). The results presented might then be tested by future research in the context of actual excerpts of the piano repertoire. In addition, the middle finger was considered in this study as a non-articulated segment starting at the metacarpophalangeal joint due to sporadic occlusions of finger markers caused by the grand piano fallboard. This modeling choice is supported by the findings of Goebl and Palmer (2013), where the metacarpophalangeal joint was identified as the finger joint that contributed the most to the vertical fingertip motion in piano performance. Capture of fingers’ motion when using a grand piano remains an important experimental challenge, particularly when using marker models that integrate lower-body and upper-body segments. The use of grand pianos when studying the biomechanics of piano performance is necessary to ensure a higher level of ecological validity in the case of both simple performance tasks or actual excerpts of the repertoire. Future studies might address this limitation by refining both the motion capture setup and data processing techniques dealing with finger marker occlusion. Furthermore, depending on the object of study, researchers might bypass this experimental challenge by addressing the impact of whole-body motion and other performance parameters on hand instead of finger kinematics.

## Conclusion

This study highlights that not only the choice of touch and articulation but also the use of pelvis and thorax motion can modify both upper-limb linear velocities and joint contribution to generate velocities at the hand and fingers. The results presented point to four main findings. First, while the direction of vertical joint velocities depended on touch and articulation at the sound production time-point, practically all types of keystrokes produced forward joint velocities. Second, pressed touch relied on a rapid and steady creation of segmental forward velocities to produce similarly loud tones than struck touch, which is characterized by greater finger downward velocities created by elbow joints. Third, choice of articulation (*staccato* or *tenuto*) had an impact on upper-limb linear velocities as well as on shoulder-girdle and wrist angular contributions to endpoint velocities not only after but also most importantly during the key descent. Fourth, the implication of trunk motion during keystrokes effectively increased segmental velocities. Specifically, pelvic anterior rotation appeared to have an important role in enhancing pianists’ potential to produce louder tones as it contributed to creating forward velocities of both distal and more proximal and heavier body segments.

To the best of our knowledge, this article represents the first empirical work that analyses the role of pelvis and thorax motion in the production of piano tones. Similar effects of body implication strategy were reported in struck and pressed touch as well as in *staccato* and *tenuto* articulations. Therefore, effective use of specific pelvic and thoracic movements seems to be an adaptable performance strategy which might help pianists reduce risks of PRMD and enhance motion efficiency and control in a variety of musical contexts. The reported findings challenge a commonly shared view in biomechanics and motor control studies that define pianists’ kinematic chain from the shoulder to the fingertip. They also relate to performance studies addressing musicians’ gestures by providing empirical evidence of how trunk motion might not only be associated with pianists’ expressive intentions.

## Data Availability Statement

The datasets for this article are not publicly available because of both participants privacy issues and ethical consent requirements. Requests to access the datasets should be directed to FV.

## Ethics Statement

The studies involving human participants were reviewed and approved by the Université de Montréal Ethics Committee (No. 18-086-CPER-D). The patients/participants provided their written informed consent to participate in this study.

## Author Contributions

All authors contributed to the conception and design of the study. FV and JP were responsible for the data collection; data processing was performed by FV, BM, and MB (kinematic data) and by FV and CT (sound data). FV executed the statistical analysis and wrote a first draft of the manuscript. FV and JP developed the guidelines of a discussion section relevant for both pianists and researchers. JP and MB modified and wrote specific excerpts of the manuscript. All authors contributed to manuscript revision and read and approved the submitted version.

## Conflict of Interest

The authors declare that the research was conducted in the absence of any commercial or financial relationships that could be construed as a potential conflict of interest.
